# Impact of Immediate Versus Staged Complete Revascularization on Short‐Term and Long‐Term Clinical Outcomes in Patients With Acute Coronary Syndrome and Multivessel Disease: A Systematic Review and Meta‐Analysis

**DOI:** 10.1002/clc.70011

**Published:** 2024-09-04

**Authors:** Qiufeng Jia, Ankai Zuo, Chengrui Zhang, Danning Yang, Yu Zhang, Jing Li, Fengshuang An

**Affiliations:** ^1^ State Key Laboratory for Innovation and Transformation of Luobing Theory Jinan China; ^2^ Key Laboratory of Cardiovascular Remodeling and Function Research, Chinese Ministry of Education Chinese National Health Commission and Chinese Academy of Medical Sciences Jinan China; ^3^ Department of Cardiology Qilu Hospital of Shandong University Jinan China; ^4^ Department of Rehabilitation Medicine The Affiliated Hospital of Qingdao University Qingdao Shandong China

**Keywords:** acute coronary syndrome, complete revascularization, immediate, meta‐analysis, multivessel, staged

## Abstract

**Background:**

In patients with acute coronary syndrome (ACS) and multivessel disease (MVD), complete revascularization (CR) improves prognosis. This meta‐analysis, summarizing recent RCTs, contrasts short‐term and long‐term clinical outcomes between immediate complete revascularization (ICR) and staged complete revascularization (SCR).

**Methods:**

We systematically searched the online database and eight RCTs were involved. The primary outcomes included long‐term unplanned ischemia‐driven revascularization, re‐infarction, combined cardiovascular (CV) death or myocardial infarction (MI), all‐cause death, CV death, stroke, and hospitalization for heart failure (HHF). The secondary outcomes were 1‐month unplanned ischemia‐driven revascularization, re‐infarction, all‐cause death, and CV death. Safety endpoints included stent thrombosis and major bleeding.

**Results:**

Eight RCTs comprising 5198 patients were involved. ICR reduced long‐term unplanned ischemia‐driven revascularization (RR 0.64, 95% CI 0.51–0.81, *p* < 0.001), combined CV death or MI (HR 0.51, 95% CI 0.34–0.78, *p* = 0.002), and re‐infarction (RR 0.66,95% CI 0.48 to 0.91, *p* = 0.012) compared with SCR. ICR also decreased 1‐month unplanned ischemia‐driven revascularization (RR 0.41, 95% CI: 0.21–0.77, *p* = 0.006) and re‐infarction (RR 0.33, 95% CI:0.15–0.74, *p* = 0.007) but increased 1‐month all‐cause death (RR 2.22, 95% CI 1.06–4.65, *p* = 0.034).

**Conclusion:**

In ACS patients with MVD, we first found that ICR significantly lowered the risk of both short‐term and long‐term unplanned ischemia‐driven revascularization and re‐infarction, as well as the long‐term composite outcome of CV death or MI compared with SCR. However, there may be an increase in 1‐month all‐cause death in the ICR group.

## Graphical abstract

Meta‐analysis of 8 RCTs (5198 patients) revealed that immediate complete revascularization (ICR) in acute coronary syndrome (ACS) with multivessel disease reduced short and long‐term unplanned ischemia‐driven revascularization and re‐infarction risks, and long‐term cardiovascular (CV) death or myocardial infarction (MI), compared to staged complete revascularization (SCR), with a potential short‐term mortality increase.

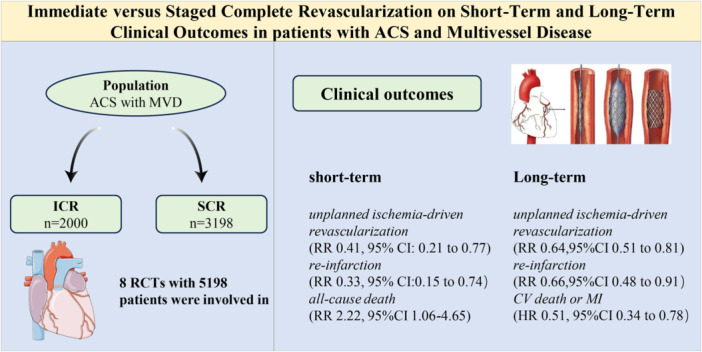

## 1 Introduction

Acute coronary syndrome (ACS) remains a leading cause of morbidity and mortality worldwide, despite substantial progress in diagnosis and treatment over the past decade [1]. Percutaneous coronary intervention (PCI) has emerged as the preferred method for re‐establishing blood flow in the culprit artery in ACS patients. A crucial observation is that Multivessel Disease (MVD) is present in approximately 50% of patients undergoing primary PCI (PPCI), a condition associated with poorer prognoses compared to single‐vessel disease [2, 3]. Previous studies have consistently demonstrated that complete coronary revascularization offers improved long‐term cardiac prognosis and enhances left ventricular function in ACS patients without cardiogenic shock [4]. In recent years, a plethora of randomized controlled trials (RCTs) and meta‐analyses have underscored the superiority of complete revascularization (CR) over PCI targeting only the culprit lesion in patients with acute ST‐segment elevation myocardial infarction (STEMI) accompanied by multivessel coronary artery disease. This approach not only reduces cardiovascular (CV) mortality, myocardial infarction (MI) incidence, and the need for subsequent revascularizations, but also augments left ventricular function [5, 6, 7, 8]. Furthermore, observational studies and meta‐analyses focusing on non‐randomized cohorts indicate that CR is linked to reduced mortality and major adverse cardiovascular events (MACE) in patients presenting with non‐ST segment elevation myocardial infarction (NSTEMI) and MVD compared to PCI limited to the infarct‐related artery (IRA) [9, 10]. Consequently, current guidelines advocate for CR in patients presenting with ACS and multivessel coronary artery disease, provided they are not experiencing cardiogenic shock [11].

Two distinct strategies for CR are available: immediate complete revascularization (ICR), which involves treating both the culprit and non‐culprit lesions simultaneously during the index procedure, and staged complete revascularization (SCR), where only the culprit lesion is addressed, followed by revascularization of non‐infarct‐related arteries (non‐IRAs) either later during the index hospitalization or in subsequent hospital admission. However, the optimal timing for non‐IRAs revascularization in ACS remains a subject of debate due to the lack of large‐scale randomized trials with a superiority design comparing these two approaches.

In light of the recent emergence of two pivotal RCTs BIOVASC [12] and MULTISTARS AMI [13], this systematic review and meta‐analysis has been conducted. It focuses on comparing the short‐term (1‐month) and long‐term clinical outcomes between two CR strategies in hemodynamically stable patients with ACS who present with multivessel coronary artery disease.

## 2 Methods

We developed a protocol that was submitted to PROSPERO and registered with the number CRD42023492019. This systematic review and meta‐analysis followed the recommendations of the Preferred Reporting Items for Systematic Reviews and Meta‐analyses (PRISMA) statement [14]. The entire process of our meta‐analysis abides by PICOS criteria.

### 2.1 Data Sources and Search Methods

We comprehensively searched the following online databases: PubMed, Embase, Cochrane Library, Web of Science, and ClinicalTrials.gov from the establishment of the databases till 25 September 2023 using the following keywords: “Myocardial Infarction,” “complete revascularization,” “percutaneous coronary intervention,” “staged,” “immediate.” A detailed search strategy is shown in Supporting Information S1: Table 1. To ensure no relevant publications were overlooked, we also manually searched for qualifying publications in the reference lists of eligible articles. Only RCTs could be involved. Two searchers conducted literature searches independently and resolved problems through discussion, seeking help and advice from third parties if necessary.

### 2.2 Inclusion Criteria

The studies included in our meta‐analysis must meet all of the following criteria: (1) the language of inclusion was English and the participants were human; (2) involved the ACS population with MVD; (3) compared ICR with SCR; (4) reported any of the following outcomes: unplanned ischemia‐driven revascularization, re‐infarction, all‐cause death, CV death, stroke, hospitalization for heart failure (HHF), CV death or MI, stent thrombosis, and bleeding; (5) The follow‐up time of the trial was at least 1 month; (6) The number of participants in the experiment should not be less than 100; (7) The study type was limited to RCTs. Studies in which participants were in cardiogenic shock were excluded.

### 2.3 Data Collection and Quality Assessment

Two reviewers (Q.J. and C.Z.) independently extracted data of interest using an electronic data collection form designed precisely. The extracted data included: (1) Baseline characteristics of included studies: trial name and number, first author, study design, publication time, Intervention methods, and follow‐up duration; (2) Characteristics of the population included in each study: gender, age, LVEF, blood pressure, SYNTAX Score, and so on; (3) Study outcomes: The primary efficacy outcomes included long‐term unplanned ischemia‐driven revascularization, re‐infarction, all‐cause death, CV death, stroke, HHF, and combined CV death or MI. The secondary outcomes were short‐term (1‐month) occurrences of unplanned ischemia‐driven revascularization, re‐infarction, all‐cause death, and CV death. The safety endpoints included stent thrombosis at both long‐term and 1‐month intervals, as well as long‐term major bleeding.

Two reviewers independently assessed the methodological quality of the included studies using the Cochrane Risk of Bias Tool 2 (Rob 2) [15]. Rob 2 is structured into five bias domains, including bias arising from the randomization process, bias due to deviations from intended interventions, bias due to missing outcome data, bias in the measurement of the outcome, and bias in the selection of the reported result.

### 2.4 Statistical Analysis

This meta‐analysis was performed using Stata17.0. For the composite outcome of CV death or MI, we extracted hazard ratios (HRs) and corresponding 95% confidence intervals (95% CIs) to make a pooled estimate. For other dichotomous outcomes, risk ratios (RRs) were used to measure the intervention effects. Statistical heterogeneity was assessed using the *χ* ^2^ test (*p* < 0.10 was considered statistically significant for heterogeneity) and was quantified using the *I* ^2^ statistic (*I* ^2^ > 50% was considered substantial heterogeneity). When *I* ^2^ < 50%, the fixed‐effects model was used to calculate the pooled effect, and when *I* ^2^ ≥ 50%, the random‐effects model was employed. Results were considered statistically significant only when *p* < 0.05. Sensitivity analysis was conducted by sequentially excluding each included study to identify potential sources of heterogeneity. Subgroup analyses were conducted based on the main characteristics of the population, dividing it into STEMI patients and NSTEMI patients. Meta‐regression was employed to assess the impact of age and gender on the relationship between CR and primary outcomes.

## 3 Results

**Figure 1:**
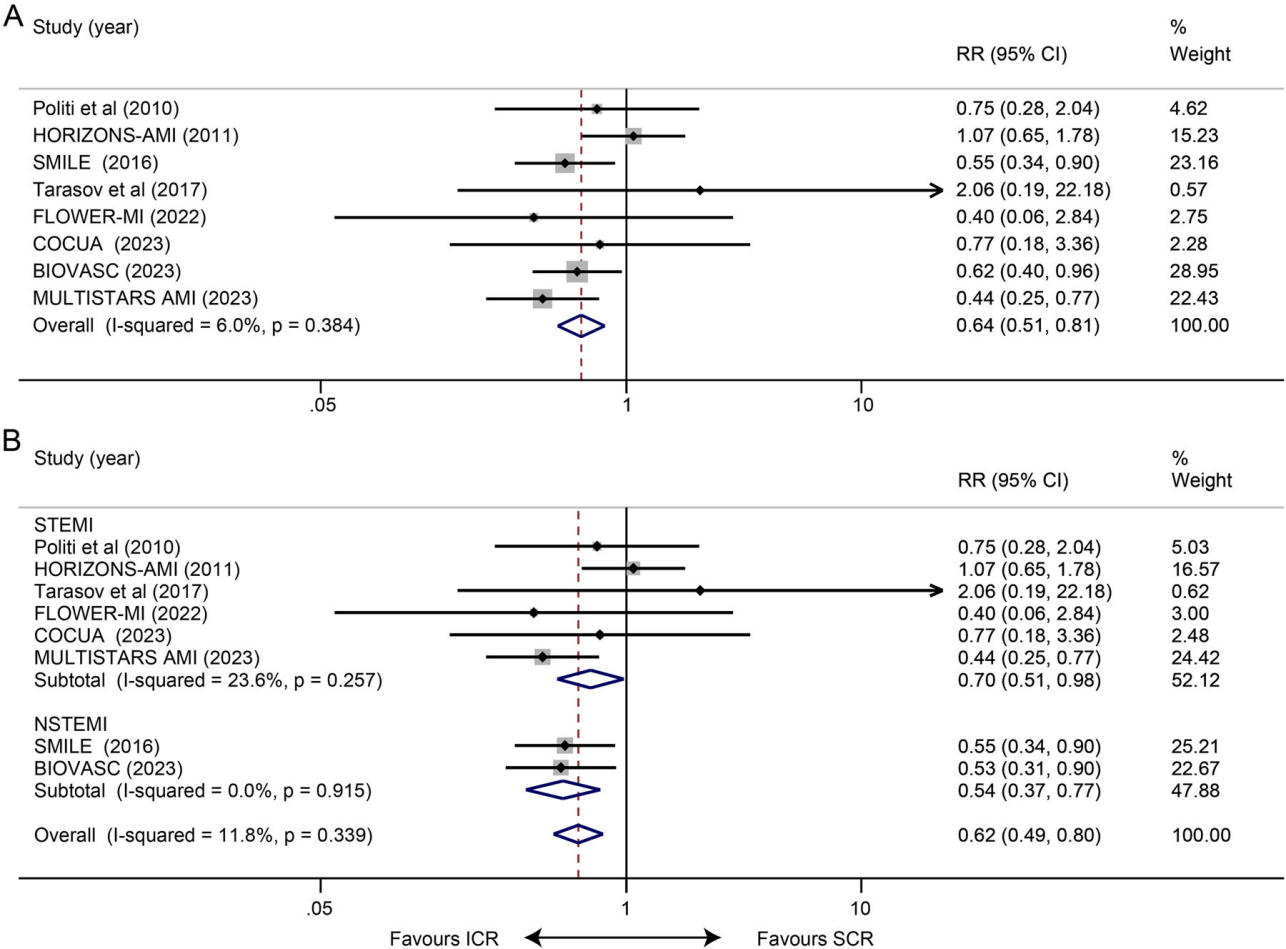
Impact of ICR versus SCR on long‐term unplanned ischemia‐driven revascularization. (A) Meta‐analysis for the risk of long‐term unplanned ischemia‐driven revascularization and (B) subgroup analyses according to STEMI or NSTEMI group.

According to the search strategy, a total of 5293 articles were searched and recorded. After scanning the titles and abstracts, 5257 irrelevant reports were excluded. The full text of the remaining records was read. Finally, 8 RCTs were included in the final analysis. The selection process is shown in Supporting Information S1: Figure 1. A total of 5198 patients were included, of which 2000 (38%) underwent immediate revascularization of non‐culprit arteries and 3198 (62%) staged revascularization. There were six STEMI trials [13, 16, 17, 18, 19, 20] (1636 patients), one NSTEMI trial [21] (1636 patients), and one ACS trial [12] (1525 patients). The characteristics of included studies and patients are shown in Table 1. The average follow‐up time was 1.2 years. The characteristics of the included population are shown in Supporting Information S1: Table 2.

**Table 1:** Baseline characteristics of included studies.

Clinical trial	Published year	Author	Follow‐up	Study design	Total patients	No of patient (ICR/SCR)	Intervention	Time interval between the first and second PCI (days)	Inclusion criteria	Endpoints^a^
Politi et al.	2010	Politi	2.5 years	RCT	130	65/65	ICR vs. SCR	56.8 ± 12.9	Patients with STEMI and multivessel CAD	1, 2, 3, 4
HORIZONS‐AMI NCT00433966	2011	Kornowski	1 year	RCT	668	275/393	ICR vs. SCR	29.0 ± 33.4	Patients with STEMI and multivessel disease	1, 2, 3, 4, 7, 13, 14
SMILE NCT01478984	2016	Sardella	1 year	RCT	527	264/263	ICR vs. SCR	4.76 ± 1.23	Patients with NSTEMI and multivessel CAD	1, 2, 3, 4, 7, 8, 10, 11
Tarasov et al. NCT01781715	2017	Tarasov	1 year	RCT	136	67/69	ICR vs. SCR	10.1 ± 5.1	Patients with STEMI and multivessel CAD	1, 2, 3, 4, 9, 10, 12, 13
FLOWER‐MI	2022	Tea	1 year	RCT	1163	44/1119	ICR vs. SCR	2.7 ± 2.4	Patients with STEMI and multivessel disease	1, 2, 3, 6, 13
COCUA NCT01180218	2023	Park	1 year	RCT	209	103/106	ICR vs. SCR	4.4 ± 7.0	Patients with STEMI and multivessel CAD	1, 2, 3, 4, 7, 8, 9, 10, 11, 12, 13
BIOVASC NCT03621501	2023	Diletti	1 year	RCT	1525	764/761	ICR vs. SCR	15.7 ± 17.8	Patients with ACS and multivessel CAD	1, 2, 3, 5, 8, 9, 10, 11, 12, 13, 14
MULTISTARS AMI NCT03135275	2023	Stahli	1 year	RCT	840	418/422	ICR vs. SCR	19–45	Patients with STEMI and multivessel CAD	1, 2, 3, 4, 5, 6, 7, 14

Abbreviations: CAD, coronary artery disease; ICR, immediate complete revascularization; NSTEMI, non‐ST segment elevation myocardial infarction; PCI, percutaneous coronary intervention; RCT, randomized control trial; SCR, staged complete revascularization; STEMI, ST‐segment elevation myocardial infarction.

a *Endpoints*: 1, unplanned ischemia‐driven revascularization; 2, re‐infarction; 3, all‐cause death; 4, CV death; 5, CV death or MI; 6, HHF; 7, stroke; 8, 1‐month unplanned ischemia‐driven revascularization; 9, 1‐month re‐infarction; 10, 1‐month all‐cause death; 11, 1‐month CV death; 12, 1‐month stent thrombosis; 13, stent thrombosis; 14, major bleeding.

**Figure 2:**
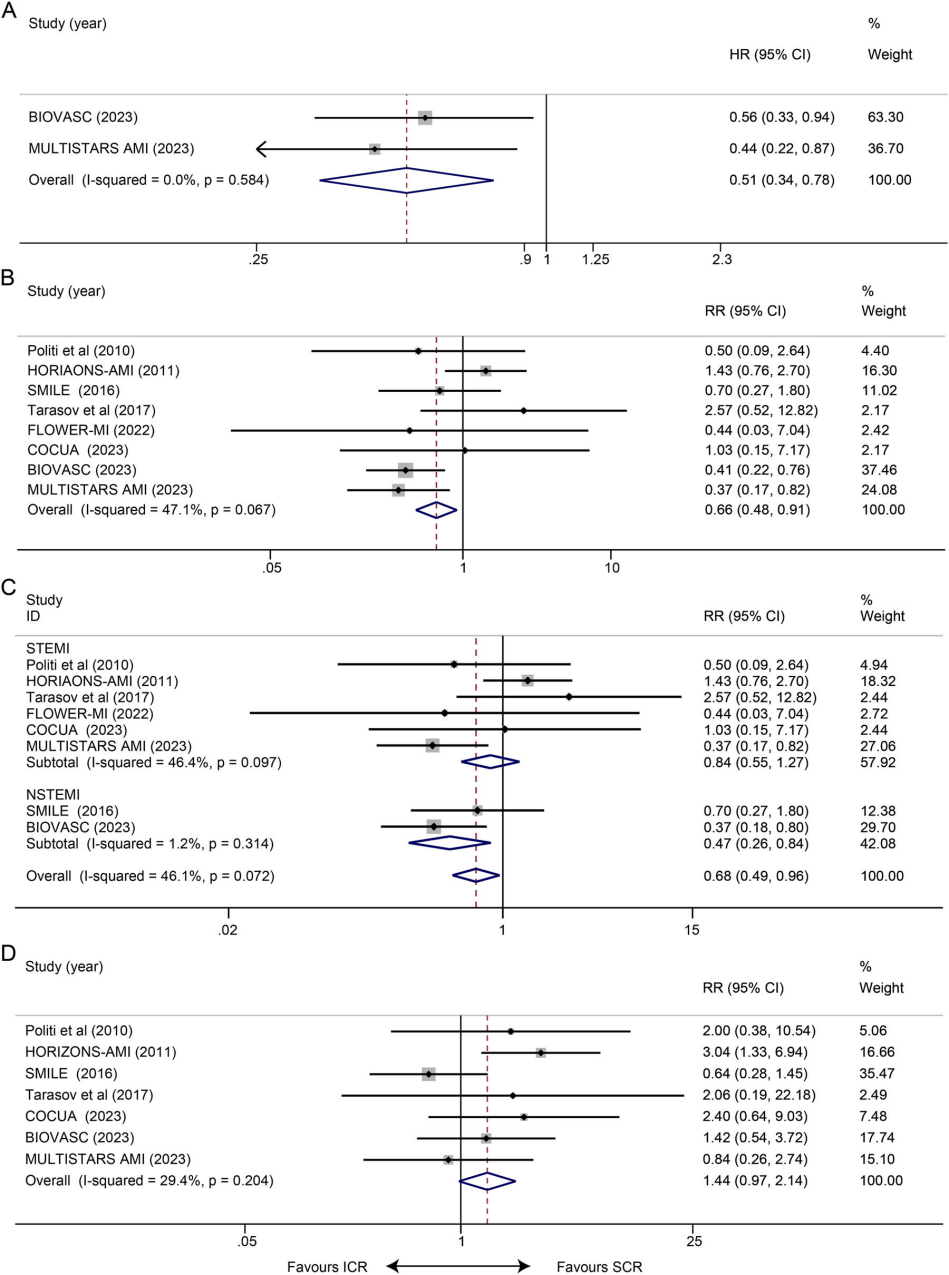
Impact of ICR versus SCR on long‐term clinical outcomes. (A) CV death or MI; (B) re‐infarction; (C) subgroup analyses of re‐infarction rates according to STEMI or NSTEMI group; and (D) CV death.

Quality assessment is shown in Supporting Information S1: Figure 2A,B. Eight trials showed some concerns because participants who underwent different procedures cannot be blinded.

### 3.1 The Primary Outcomes

#### 3.1.1 Unplanned Ischemia‐Driven Revascularization

Eight studies reported the occurrence of unplanned ischemia‐driven revascularization at long term, and overall, ICR reduced the risk of unplanned ischemia‐driven revascularization compared with SCR (RR 0.64, 95% CI 0.51–0.81, *p* < 0.001; Figure 1A). No obvious statistical heterogeneity was observed between the studies (*I*² = 6.0%, *p* = 0.384). When eliminating each study, the result remained stable. Subgroup analysis illustrated a consistency no matter whether patients had STEMI or NSTEMI (STEMI: RR 0.70, 95% CI 0.51–0.98, *p* = 0.035; *I*² = 23.6%, *p* = 0.257; NSTEMI: RR 0.54, 95% CI 0.37–0.77, *p* = 0.001; *I*² = 0.0%, *p* = 0.915; Figure 1B).

#### 3.1.2 CV Death or MI

Two studies reported statistics to do with CV death or MI at long term. Meta‐analysis showed that treating with ICR decreased the incidence of the composite outcome (HR 0.51, 95% CI 0.34–0.78, *p* = 0.002; Figure 2A). Statistical heterogeneity between the studies was not present (*I*² = 0.0%, *p* = 0.584). The result remained stabilized when we eliminated each study one by one.

Eight studies reported the occurrence of re‐infarction at long term, and ICR reduced the incidence of re‐infarction compared with SCR (RR 0.66, 95% CI 0.48–0.91, *p* = 0.012; Figure 2B). Statistical heterogeneity between the studies was not present (*I*² = 47.1%, *p* = 0.067). The subgroup analysis indicated a significant reduction in re‐infarction rates with ICR compared to SCR in NSTEMI patients (RR 0.47, 95% CI 0.26–0.84, *p* = 0.011; *I*² = 1.2%, *p* = 0.314), but not in STEMI patients (RR 0.84, 95% CI 0.55–1.27, *p* = 0.405; *I*² = 46.4%, *p* = 0.097) (Figure 2C). Eight studies reported data on CV death at long term. However, we did not observe a significant difference in CV mortality (RR: 1.44, 95% CI 0.97–2.14, *p* = 0.068; *I*² = 29.4%, *p* = 0.204; Figure 2D).

#### 3.1.3 All‐Cause Death, Stroke, and HHF

**Figure 3:**
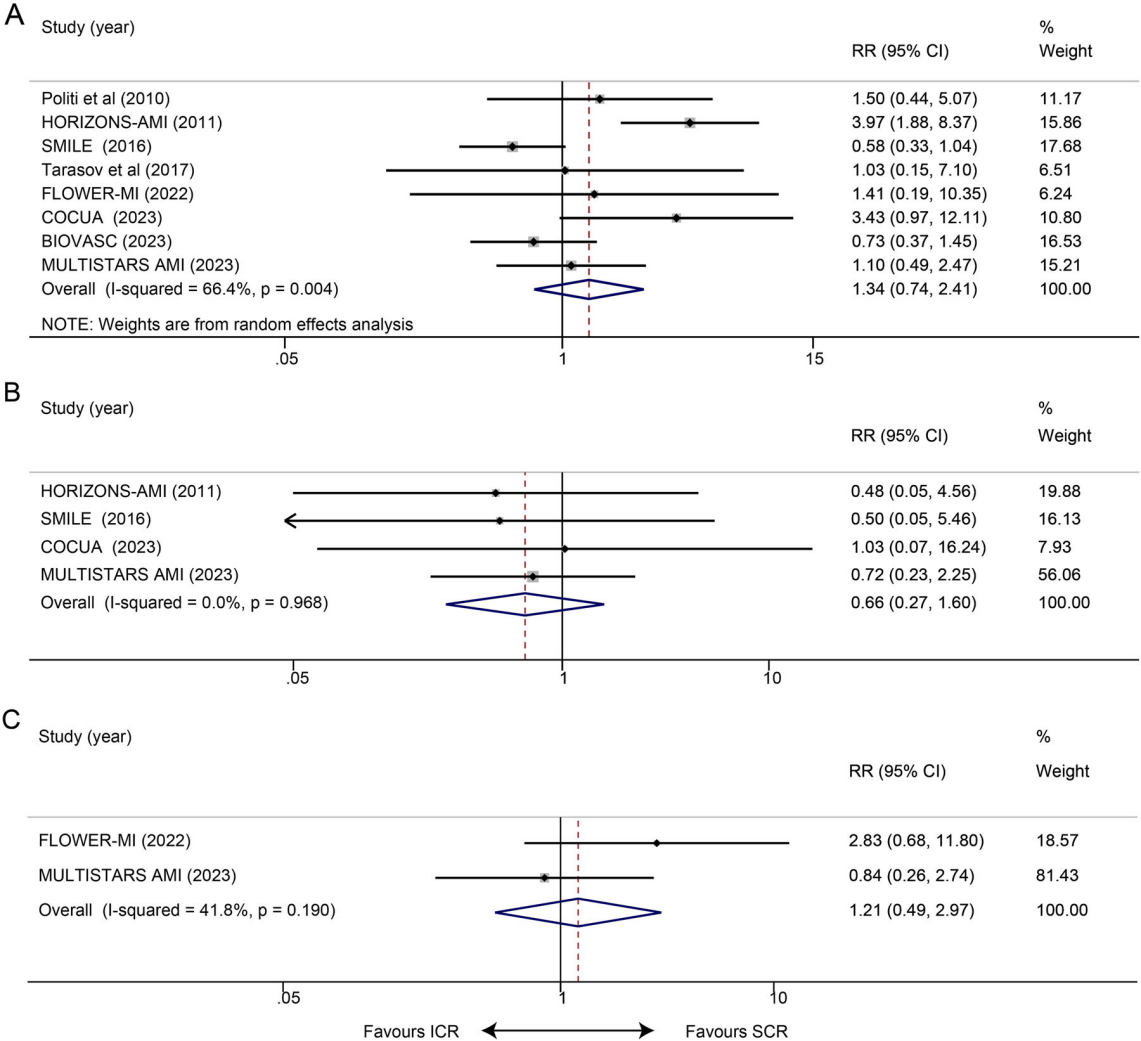
Impact of ICR versus SCR on long‐term clinical outcomes. (A) All‐cause death; (B) stroke; and (C) HHF.

Seven studies reported the occurrence of all‐cause death at long term. The results indicated that ICR showed no advantage in reducing all‐cause mortality (RR: 1.34, 95% CI 0.74–2.41, *p* = 0.339; *I*² = 66.4%, *p* = 0.004; Figure 3A). Four studies reported the incidence of long‐term stroke among participants, and there was no significant difference in stroke among patients in the ICR group compared with those in the SCR group (RR 0.66, 95% CI 0.27–1.60, *p* = 0.357; *I*² = 0.0%, *p* = 0.968; Figure 3B). Two trials worked on long‐term HHF, and there was no difference between ICR and SCR as regards the risk of HHF (RR 1.21, 95% CI 0.49–2.97, *p* = 0.667; *I*² = 41.8%, *p* = 0.190; Figure 3C).

### 3.2 The Secondary Outcomes

**Figure 4:**
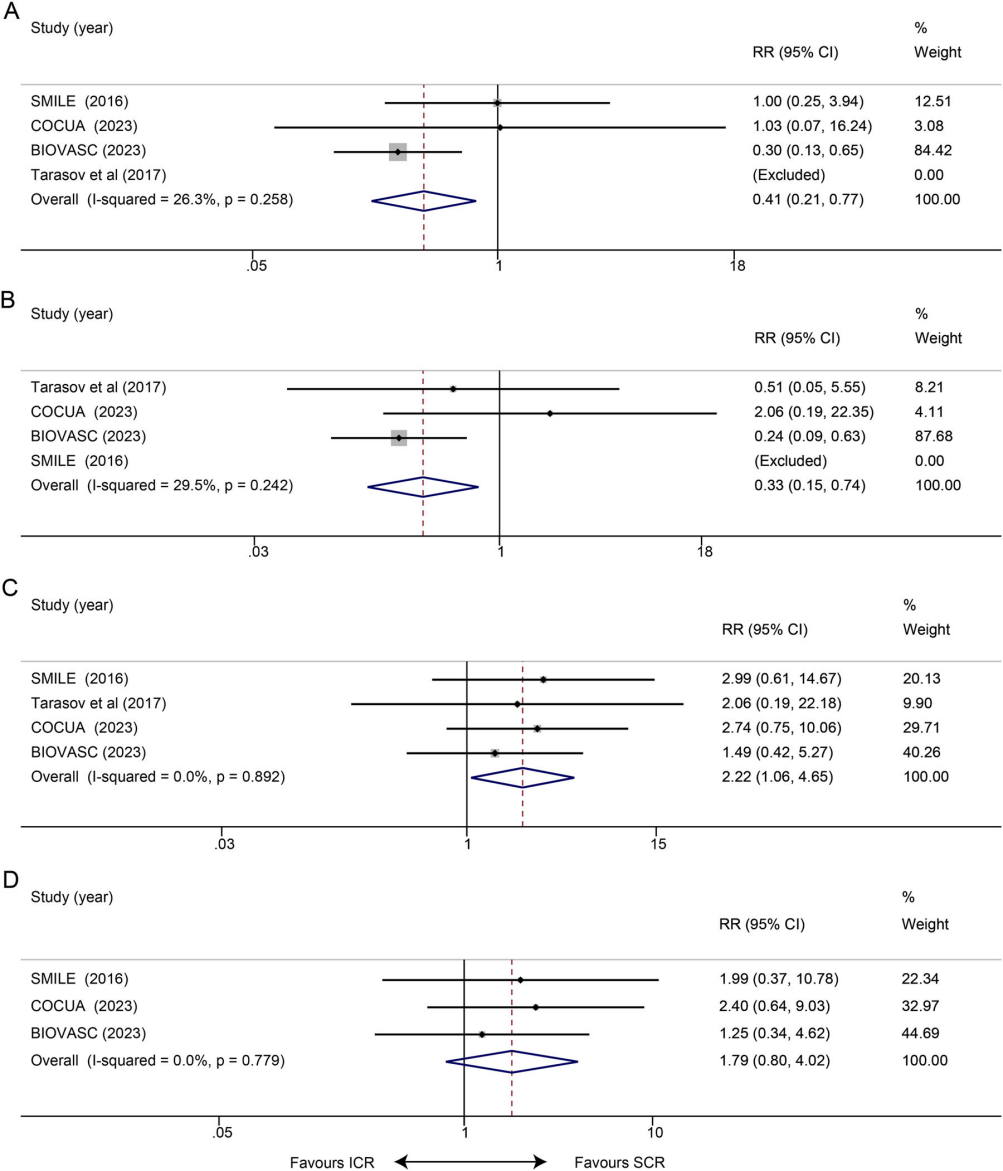
Impact of ICR versus SCR on short‐term (1‐month) clinical outcomes. (A) Unplanned ischemia‐driven revascularization; (B) re‐infarction; (C) all‐cause death; and (D) CV death.

Three studies reported unplanned ischemia‐driven revascularization and re‐infarction at 1 month. Meta‐analysis indicated that ICR showed significant effects in the two aspects compared with SCR (unplanned ischemia‐driven revascularization: RR 0.41, 95% CI 0.21–0.77, *p* = 0.006; *I*² = 26.3%, *p* = 0.258; Figure 4A; re‐infarction: RR 0.33, 95% CI 0.15–0.74, *p* = 0.007; *I*² = 29.5%, *p* = 0.242; Figure 4B). Data on 1‐month all‐cause deaths were reported in four studies, and the results showed that ICR increased the risk of 1‐month all‐cause mortality compared with SCR (RR 2.22, 95% CI 1.06–4.65; *p* = 0.034; *I*² = 0.0%, *p* = 0.892; Figure 4C). When we excluded each study individually, the results held steady. Three studies reported the occurrence of CV death at 1 month, and there was no statistically significant difference between patients in the ICR group and those in the SCR group (RR 1.79, 95% CI 0.80–4.02, *p* = 0.165; *I*² = 0.0%, *p* = 0.779; Figure 4D).

### 3.3 The Safety Endpoints

Five studies provided accessible statistics associated with Stent thrombosis, and treating with ICR did not increase the risk of 1‐month (RR 1.21, 95% CI 0.53–2.78, *p* = 0.651; *I*² = 0.0%, *p* = 0.613; Supporting Information S1: Figure 3A) and long‐term (RR 1.76, 95% CI 0.99–3.15, *p* = 0.055; *I*² = 9.7%, *p* = 0.351; Supporting Information S1: Figure 3B) stent thrombosis compared with SCR. Three studies reported long‐term major bleeding. We could not observe a significant statistical difference in the ICR group compared with the SCR group (RR 1.09, 95% CI 0.62–1.92, *p* = 0.756; *I*² = 50.7%, *p* = 0.132; Supporting Information S1: Figure 3C).

### 3.4 Meta‐Regression

Meta‐regression analysis demonstrated no linear relationship between age and the incidence of unplanned ischemia‐driven revascularization (RR 0.94, 95% CI 0.83–1.06, *p* = 0.243; Supporting Information S1: Figure 4A), nor was there a linear association between male ratio and the incidence of unplanned ischemia‐driven revascularization (RR 0.98, 95% CI 0.80–1.19, *p* = 0.77; Supporting Information S1: Figure 4B). Additionally, meta‐regression revealed no significant modifying effect of age (RR 0.90, 95% CI 0.72–1.11, *p* = 0.257; Supporting Information S1: Figure 4C) or male ratio (RR 0.93, 95% CI 0.77–1.13, *p* = 0.394; Supporting Information S1: Figure 4D) on the meta‐analysis results for re‐infarction.

## 4 Discussion

The meta‐analysis, encompassing 8 RCTs with 5198 participants, first revealed the impact of ICR versus SCR on both short‐term (1‐month) and long‐term clinical outcomes in ACS patients with MVD. As opposed to SCR, ICR treatment demonstrated a marked reduction in the incidence of unplanned ischemia‐driven revascularization, the composite outcome of CV death or MI, and re‐infarction over the long term. Subgroup analyses further demonstrated that ICR reduced the risk of long‐term unplanned ischemia‐driven revascularization in both STEMI and NSTEMI, but only reduced the risk of long‐term re‐infarction in NSTEMI. Moreover, ICR exhibited a beneficial effect in diminishing short‐term (1‐month) events related to unplanned ischemia‐driven revascularization and re‐infarction. However, there was a slight increase in all‐cause mortality at 1 month with ICR. In addition, ICR was deemed relatively safe, showing no significant increase in the risk of major bleeding and stent thrombosis risks. Finally, meta‐regression revealed no linear relationship between age or gender with long‐term repeat revascularization or re‐infarction.

Over the past decade, numerous RCTs have consistently demonstrated that CR significantly reduces the risk of CV death, recurrent MI, and the necessity for future revascularization in hemodynamically stable patients with STEMI and multivessel coronary artery disease. Importantly, this benefit is achieved without an increased risk of bleeding, stroke, or contrast‐associated nephropathy [5, 6, 7, 8, 22]. While dedicated RCTs directly comparing CR with IRA‐only PCI in NSTEMI patients are lacking, observational studies and meta‐analyses of non‐randomized studies have suggested significant advantages of CR [9, 10]. However, the optimal timing for CR remains controversial. This can be largely attributed to the lack of large‐scale RCTs directly comparing these two CR strategies. The SMILE trial, the first RCT found that ICR may be superior to SCR, demonstrating a reduction in the incidence of major adverse cardiovascular and cerebrovascular events (MACCE) and repeat coronary revascularization with ICR. Nonetheless, the majority of RCTs have indicated that simultaneous treatment of non‐IRAs and staged revascularization yielded similar clinical outcomes. Most meta‐analyses incorporated studies that contrasted immediate or staged CR with culprit‐only revascularization, with subsequent network or pairwise analyses, or included predominantly non‐RCTs. These meta‐analyses have yielded divergent conclusions. Specifically, two meta‐analyses, primarily based on non‐RCTs, concluded that ICR elevated the risk of mortality both at 30‐day and long‐term intervals [23, 24]. Conversely, an analysis focusing exclusively on four RCTs reported a reduced risk of unplanned ischemia‐driven revascularization at the longest follow‐up for patients undergoing single‐stage CR [25]. Feng Yujia performed a pairwise and network meta‐analysis including 17 RCTs further confirming that ICR reduced MACE and unplanned ischemia‐driven revascularization compared with SCR [26]. Some studies have documented similar clinical outcomes between ICR and SCR [27, 28, 29]. Fortunately, the recent publication of several large‐scale RCTs has shed new light on this contentious topic, providing robust evidence to inform the optimal revascularization strategy. Notably, the BIOVASC trial demonstrated that ICR was non‐inferior to SCR for the primary composite outcome, with an associated reduction in MI and unplanned ischemia‐driven revascularization. Similarly, the MULTISTARS AMI trial indicated that immediate multivessel PCI held superiority over staged multivessel PCI in terms of reducing the risk of nonfatal MI and unplanned ischemia‐driven revascularization. In light of these developments, our meta‐analysis, incorporating only RCTs, aims to directly compare the short‐term (1‐month) and long‐term clinical outcomes of these two CR strategies, in the hope of informing the choice of the best revascularization strategy.

Based on long‐term outcomes, our study found that (a) compared with SCR, ICR reduced the risk for unplanned ischemia‐driven revascularization by 36%, and subgroup analyses showed consistency irrespective of whether patients presented with STEMI or NSTEMI; (b) intervening with ICR could decrease 49% relative risk of the composite outcome of CV death or MI; (c) the risk of re‐infarction was also reduced by 34%, while CV mortality remained unchanged; and (d) no significant advantage of ICR over SCR was observed in terms of long‐term all‐cause death, stroke, and HHF. It's noteworthy that the composite outcome of CV death or MI was reported in only two recent studies. These studies elucidated that ICR was linked with fewer incidents of the composite outcome, predominantly influenced by a lower rate of re‐infarction. Thus, the observed reduction in composite risk was primarily driven by decreased re‐infarction rates. These results suggest that plaque instability may not be limited to culprit lesions. Another potentially vulnerable plaque might be an already ruptured but asymptomatic plaque such as those detected by intravascular ultrasound (IVUS) at non‐culprit sites from patients undergoing intervention with an ACS, resulting in recurrent ischemia and infarction [30]. Consequently, addressing both culprit and non‐culprit vessels during the initial intervention could lead to improved long‐term clinical outcomes. In summary, the strength of ICR in improving long‐term prognosis in ACS patients with MVD was confirmed in our study. This finding provides direct evidence supporting the adoption of ICR as a therapeutic strategy in this population.

In terms of 1‐month outcomes, the advantageous impact of ICR on unplanned ischemia‐driven revascularizations and re‐infarction was significant, aligning with the findings observed in long‐term follow‐up. No substantial difference was seen in the incidence of CV death. However, we observed a 1.22‐fold elevation in the risk of all‐cause death at 1 month with ICR group. The reasons behind this increased mortality risk following multivessel intervention during the index primary PCI procedure remain elusive but are likely to be multifaceted. First, the local inflammatory response in the early phase of ACS might elevate procedural risks [31]; Second, simultaneous multivessel PCI escalates the usage of contrast material, potentially heightening mortality risks, particularly in instances of radiocontrast‐induced nephropathy [32]; Lastly, patients undergoing ICR typically experience longer operative times and more complex procedures involving both culprit and non‐culprit vessels, which might be less well‐tolerated during the perioperative period. Possibly to avoid an increase in short‐term mortality, SCR is usually taken in current clinical practice.

The safety concerns in our study primarily focused on the risks of stent thrombosis and major bleeding. Despite the well‐established direct relationship between the prethrombotic environment during the periprocedural period and the incidence of stent thrombosis [33], our findings did not reveal an elevated risk of stent thrombosis both at 1 month and long‐term post‐immediate multivessel revascularization. This aligns with the outcomes observed in the majority of the RCTs included in our analysis. Besides, ICR did not demonstrate an increased risk of long‐term bleeding complications. Consequently, based on these observations, we conclude that ICR appears to be relatively safe when compared to SCR, reinforcing its viability as a treatment option.

Furthermore, considering the unique characteristics of STEMI and NSTEMI populations [34], subgroup analyses were conducted by ACS type. ICR decreased the incidence of unplanned ischemia‐driven revascularization in both STEMI and NSTEMI patients. ICR was associated with a lower risk of re‐infarction compared to SCR in NSTEMI patients, while no significant difference was found in STEMI patients. Regrettably, data limitations prevented us from conducting broader subgroup analyses. Finally, meta‐regression based on age and gender was conducted. Fabrizio et al.'s study indicated that gender differences might introduce a bias in revascularization strategies. It revealed that women undergoing PCI with coronary stenting experienced similar cardiac event rates as men despite higher risk factors, yet faced significantly higher mortality rates in STEMI patients [35]. Nonetheless, our meta‐regression results indicated that neither age nor gender significantly influenced the meta‐analysis outcomes for revascularization and re‐infarction. This may be attributed to the consistent mean age and sex ratio across the included RCTs, along with the limited baseline data these studies provided.

This meta‐analysis stands out for its exclusive inclusion of RCTs and it is the first to evaluate both short‐term (1‐month) and long‐term outcomes of two distinct revascularization strategies. To summarize, achieving CR during the initial PCI is linked to improved long‐term cardiac outcomes in ACS patients. In particular, our study is the first to demonstrate that ICR could decrease the risk of composite outcomes of CV death or MI. Notably, we found for the first time that ICR diminishes the risk of 1‐month revascularization and re‐infarction. Moreover, the 1‐time complete PCI may also curtail future hospital stays and reduce the need for surgeries. This approach, by averting exposure to a secondary invasive procedure, inherently reduces the associated risks and expenses, thus offering favorable economic implications. Although ICR was associated with an increased 1‐month mortality risk, multivessel PCI during the index procedure could be advantageous. As yet, there are still several ongoing trials dedicated to clarifying the impact of ICR in patients with ACS, such as the IMODERN (NCT03298659) and Future (NCT05967663) Studies. The comparative prognosis of ICR versus SCR in this population awaits further confirmation through larger‐scale studies, incorporating subgroup analyses based on key population characteristics like diabetes, atrial fibrillation, heart failure, and the type of stents used.

The limitations of this meta‐analysis are as follows. First, the predominant inclusion of patients with STEMI in this study limits its applicability to individuals with NSTEMI and unstable angina. Second, the lack of blinding in the trials incorporated into this meta‐analysis introduces a potential bias. Thirdly, there was variability in the definition of MACE across the RCTs considered in this study. Consequently, MACE, which is typically a primary outcome in such trials, was not included as an observed outcome in our meta‐analysis. This inconsistency might affect the comprehensiveness of our findings. Fourth, the variability in the interval between initial and subsequent PCI across studies suggests a need for future research to ascertain if the advantage of ICR is influenced by the timing of the staged procedure in the comparison group. Finally, included statistics of reported outcomes were restricted because of the finite amount of original studies. The constraints in available data precluded the conduct of further subgroup or regression analyses, potentially limiting the depth of our conclusions.

## 5 Conclusion

Our meta‐analysis demonstrates that in ACS patients with MVD and hemodynamic stability, ICR significantly decreases the risk of both short‐term and long‐term unplanned ischemia‐driven revascularization and re‐infarction, as well as the long‐term composite outcome of CV death or MI compared with SCR. At the same time, we need to be alert to the potential increase in the risk of all‐cause mortality at 1 month associated with ICR. Finally, ICR is relatively safe, not substantially elevating the risk of major bleeding or stent thrombosis.

## Author Contributions

**Qiufeng Jia:** methodology, statistical analysis, quality assessment, and writing and polishing the draft. **Ankai Zuo:** methodology, writing, and polishing the draft. **Chengrui Zhang:** data collection and bias assessment. **Danning Yang** and **Yu Zhang:** data visualization and consultation. **Jing Li** and **Fengshuang An:** reviewing and editing the manuscript.

## Conflicts of Interest

The authors declare that the research was conducted in the absence of any commercial or financial relationships that could be construed as a potential conflict of interest.

## Supporting information

Supporting information.

## Data Availability

The data utilized in this meta‐analysis are publicly available and have been previously published.
