# Supplementary material for: Impact of Immediate Versus Staged Complete Revascularization on Short‐Term and Long‐Term Clinical Outcomes in Patients With Acute Coronary Syndrome and Multivessel Disease: A Systematic Review and Meta‐Analysis
Source: Clin Cardiol. 2024 Sep 4;47(9):e70011. doi: 10.1002/clc.70011 (PMC11372235; doi:10.1002/clc.70011)
Supplement: Supplementary file 1 — Supporting information. [file CLC-47-e70011-s001.docx]

**Supplementary Materials**

**Supplementary Table 1. Search Strategy.**

| **P** | Myocardial Infarction OR STEMI OR NSTEMI OR Myocardial Infarctions OR Infarction, Myocardial OR Infarctions, Myocardial OR Cardiovascular Stroke OR Cardiovascular Strokes OR Stroke, Cardiovascular OR Strokes, Cardiovascular OR Myocardial Infarct OR Myocardial Infarcts OR Infarct, Myocardial OR Infarcts, Myocardial OR Heart Attack OR Heart Attacks OR acute coronary syndrome OR acute coronary syndromes OR ACS OR Coronary Syndrome, Acute OR Coronary Syndromes, Acute OR Syndrome, Acute Coronary OR Syndromes, Acute Coronary OR unstable angina pectoris |
| --- | --- |
| **I** | (percutaneous coronary intervention OR PCI OR angioplasty OR revascularization) And (Multivessel OR multi-vessel OR non-culprit OR non-infarct OR staged OR immediate OR complete) |
| **S** | randomized clinical trial OR clinical trial, randomized OR trial, randomized clinical OR randomized controlled clinical trial OR controlled clinical trial, randomized |

**Supplementary Table 2. Patient characteristics at baseline.**

| **Clinical trial** | **Published year** | **Age** | **male（n,%）** | **Hypertension（n,%）** | **Diabetes mellitus（n,%）** | **LVEF(%)** | **Anterior location of MI（n,%）** | **Three-vessel disease（n,%）** | **Chronic renal failure（n,%）** | **SYNTAX Score** |
| --- | --- | --- | --- | --- | --- | --- | --- | --- | --- | --- |
| Politi et al. | 2010 | 64.3±11.4 | 102(78.5) | 74(56.9) | 21（16.2） | 45.7±9.5 | 59（45.4） | 48(36.9) | 33(25.4) | NA |
| HORIZONS-AMI NCT00433966 | 2011 | 62.9 | 537(80.4) | 16.9 | NA | 58.1 | 250(37.4) | NA | NA | NA |
| SMILE  NCT01478984 | 2016 | 70.5±12.3 | 416（78.9） | 367（69.6） | 202（38.3） | 48.2±11.2 | 471（89.4） | NA | NA | 15.9±3 |
| Tarasov et al. NCT01781715 | 2017 | 58.9±10.6 | 91(66.9) | 125(91.9) | 30(22.1) | 51.3±8.3 | NA | 63(46.3) | NA | 18.9±7.7 |
| FLOWER-MI | 2022 | 62.2±11.2 | 966(83.1) | 515(44.3) | 189(16.3) | 51.3±9.0 | 370(31.8) | NA | 23(2..0) | 15.1±8.4 |
| COCUA  NCT01180218 | 2023 | 62.7±10.6 | 107（81.3） | 104（49.8） | 79（37.8） | 51.4±11.1 | 93（44.5） | 39（18.7） | NA | NA |
| BIOVASC NCT03621501 | 2023 | 65.4±11.1 | 1187(77.8) | 818(53.6) | 321(21.0) | NA | 554(36.2) | NA | 78(5.1) | NA |
| MULTISTARS AMI NCT03135275 | 2023 | 65.0±12.7 | 662(78.8) | 440(52.3) | 131(15.6) | NA | 329(39.2) | NA | NA | NA |

Abbreviations: LVEF**,** left ventricular ejection fraction; MI, myocardial Infarction; NA, not available.

**Supplementary Figure 1** PRISMA flowchart of selection.

**
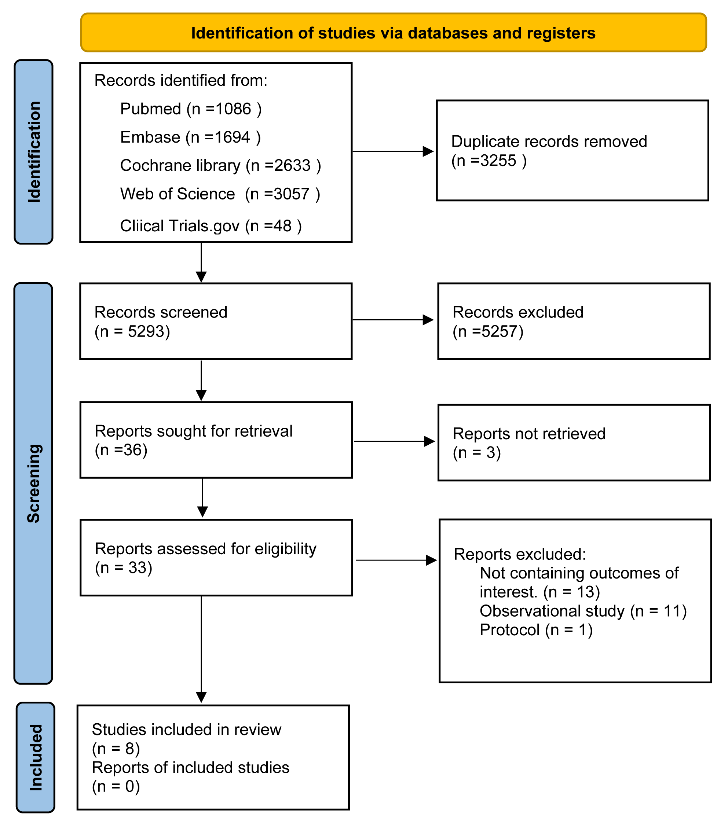
**

**Supplementary Figure 2**

(A) Risk of bias graph; (B) Risk of bias summary.

**
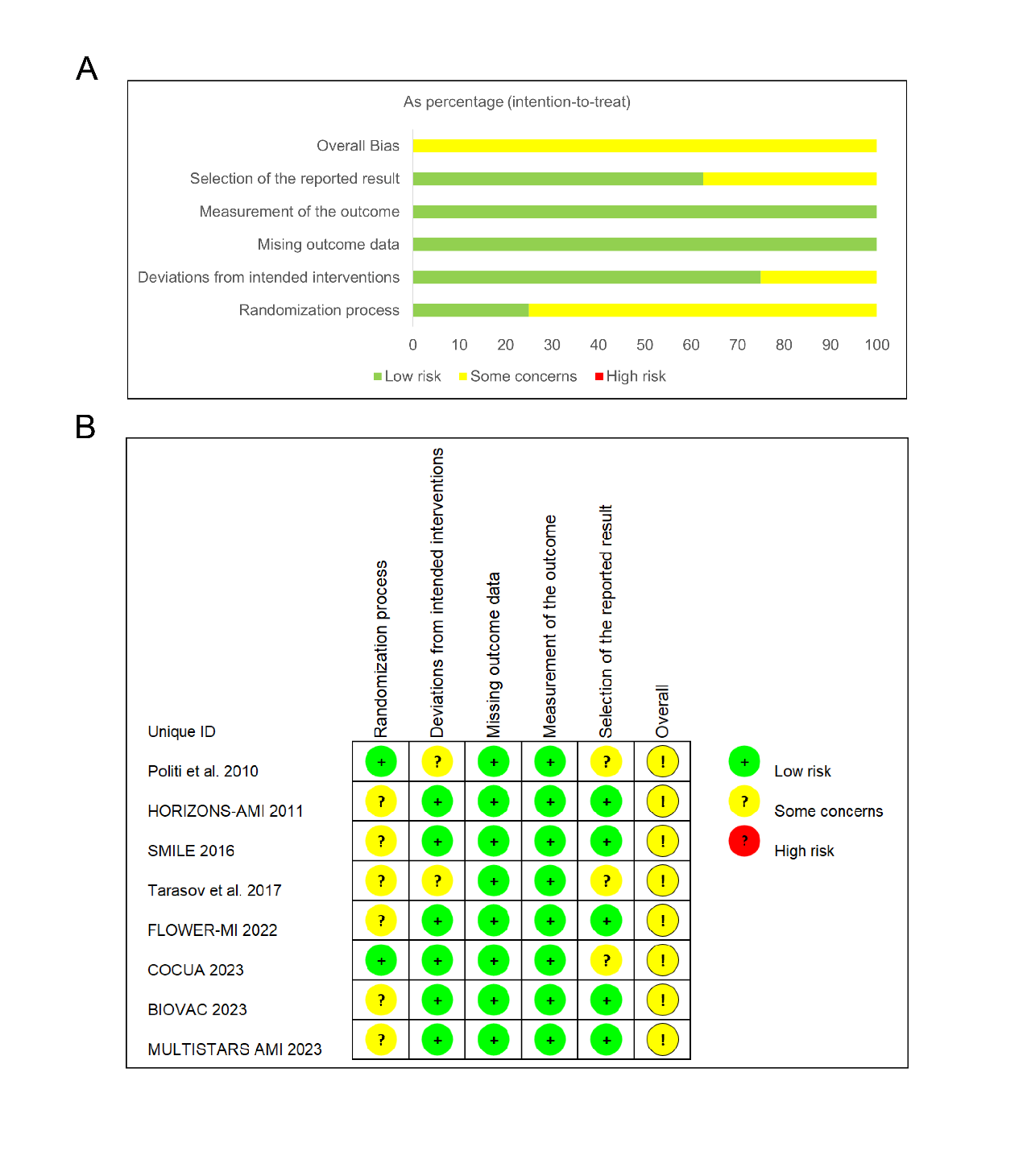
**

**Supplementary Figure 3** Impact of ICR vs. SCR on the risk of safety endpoints. (A) 1-month stent thrombosis; (B) long-term stent thrombosis; (C) long-term major bleeding.

**
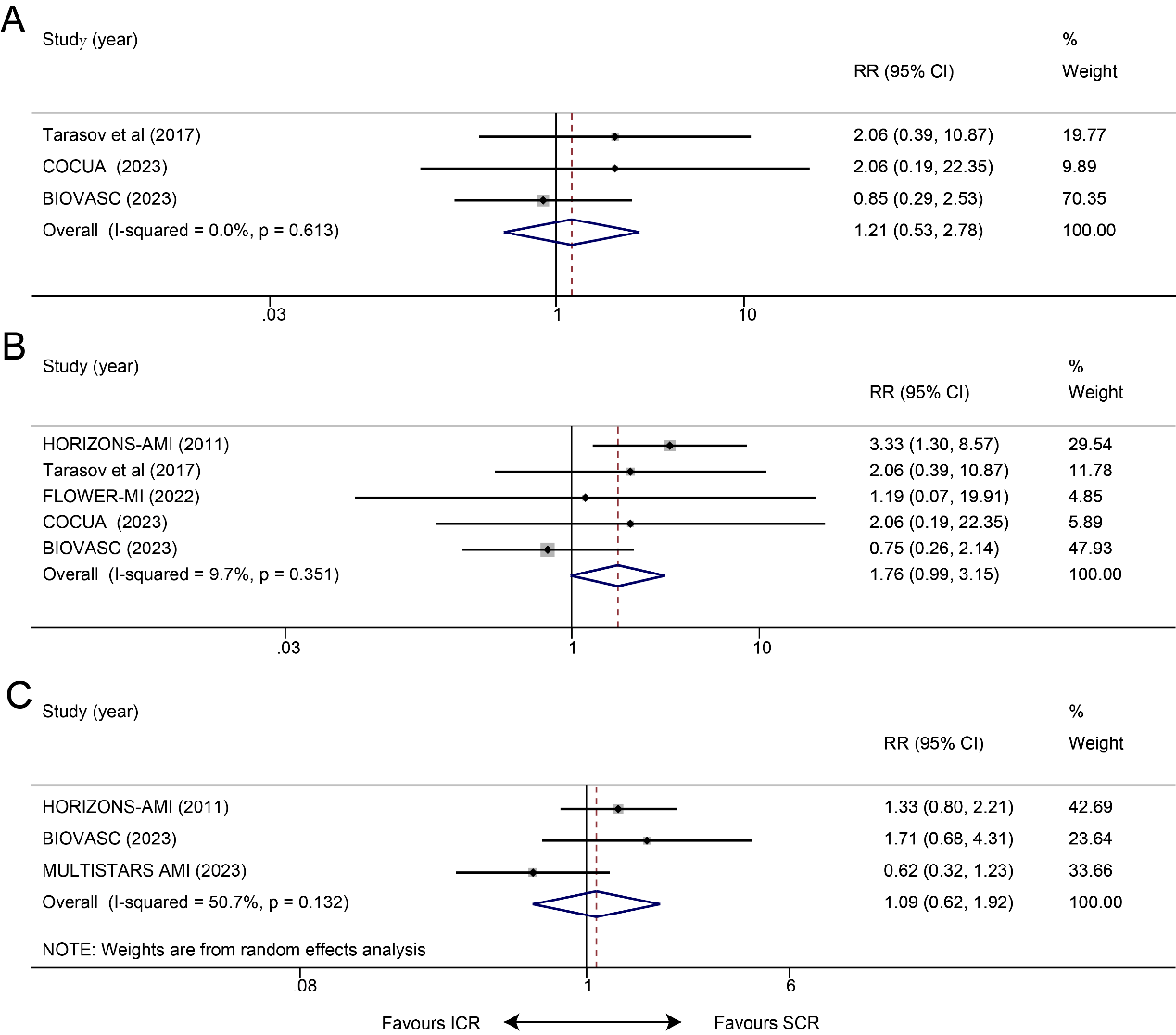
**

**Supplementary Figure 4**

Meta-regression

(A) meta-regression of age and unplanned ischemia-driven revascularization; (B) meta-regression of male ratio and unplanned ischemia-driven revascularization; (C) meta-regression of age and re-infarction; (D) meta-regression of male ratio and re-infarction.

**
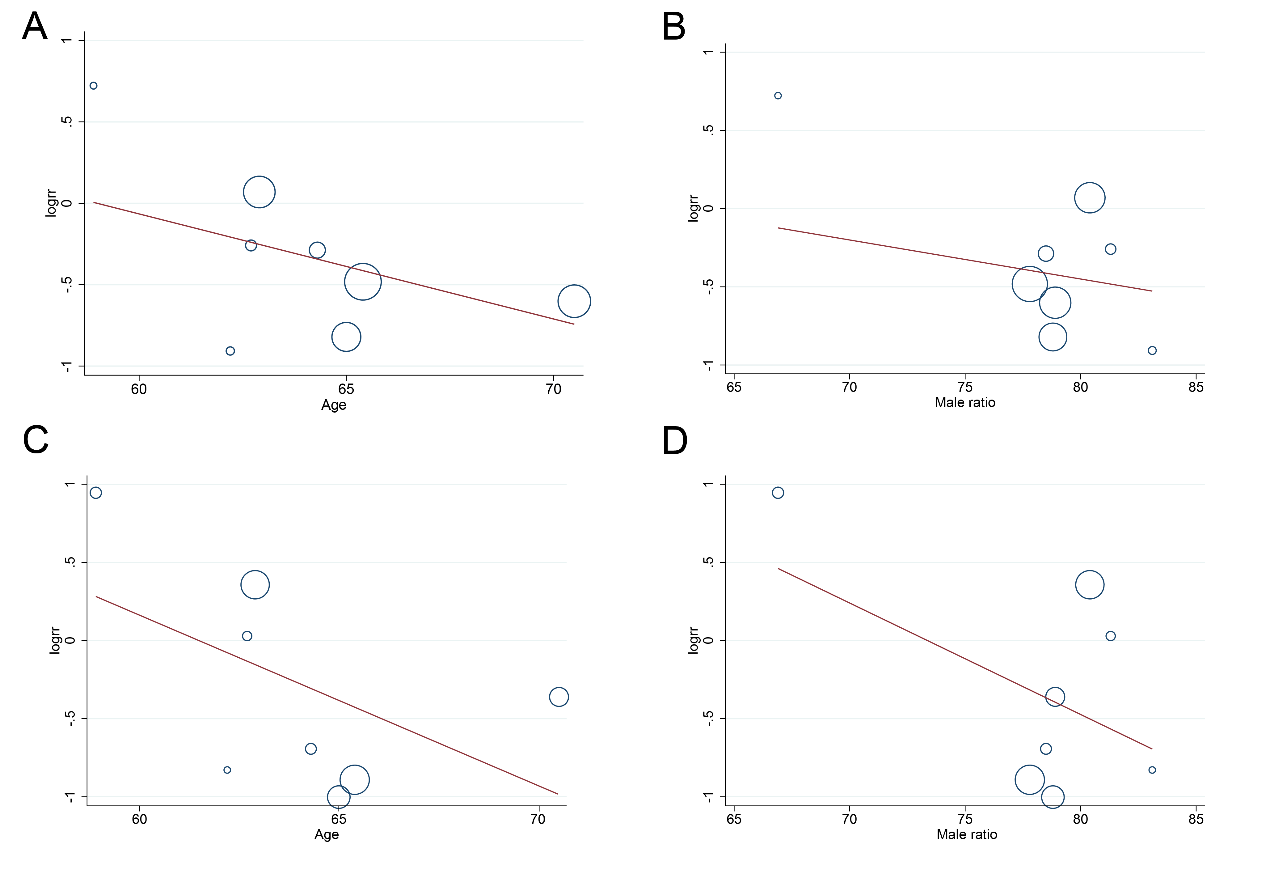
**
